# In Vitro Analysis of Deoxynivalenol Influence on Steroidogenesis in Prostate

**DOI:** 10.3390/toxins13100685

**Published:** 2021-09-26

**Authors:** Kinga Anna Urbanek, Karolina Kowalska, Dominika Ewa Habrowska-Górczyńska, Kamila Domińska, Agata Sakowicz, Agnieszka Wanda Piastowska-Ciesielska

**Affiliations:** 1 Department of Cell Cultures and Genomic Analysis, Medical University of Lodz, Zeligowskiego 7/9, 90-752 Lodz, Poland; kinga.urbanek@umed.lodz.pl (K.A.U.); karolina.kowalska1@umed.lodz.pl (K.K.); dominika.habrowska@umed.lodz.pl (D.E.H.-G.); 2 Department of Comparative Endocrinology, Medical University of Lodz, Zeligowskiego 7/9, 90-752 Lodz, Poland; kamila.dominska@umed.lodz.pl; 3 Department of Medical Biotechnology, Medical University of Lodz, Zeligowskiego 7/9, 90-752 Lodz, Poland; agata.sakowicz@umed.lodz.pl

**Keywords:** deoxynivalenol, steroidogenesis, mycotoxin, carcinogenesis, dehydroepiandrosterone

## Abstract

Deoxynivalenol (DON) is a type-B trichothecene mycotoxin produced by *Fusarium* species, reported to be the most common mycotoxin present in food and feed products. DON is known to affect the production of testosterone, follicle stimulating hormone (FSH) and luteinizing hormone (LH) in male rats, consequently affecting reproductive endpoints. Our previous study showed that DON induces oxidative stress in prostate cancer (PCa) cells, however the effect of DON on the intratumor steroidogenesis in PCa and normal prostate cells was not investigated. In this study human normal (PNT1A) and prostate cancer cell lines with different hormonal sensitivity (PC-3, DU-145, LNCaP) were exposed to DON treatment alone or in combination with dehydroepiandrosterone (DHEA) for 48 h. The results of the study demonstrated that exposure to DON alone or in combination with DHEA had a stimulatory effect on the release of estradiol and testosterone and also affected progesterone secretion. Moreover, significant changes were observed in the expression of genes related to steroidogenesis. Taken together, these results indicate that DON might affect the process of steroidogenesis in the prostate, demonstrating potential reproductive effects in humans.

## 1. Introduction

Deoxynivalenol (DON) is one of the naturally occurring mycotoxins belonging to the group of trichothecenes (type B) [1]. It is produced by *Fusarium graminearum (Gibberellazeae*) and *Fusarium culmorum* species. DON as a contaminant is mostly found in barley and wheat, but also in some processed food products such as wheat flour, bread, breakfast cereals, noodles, baby and infant foods, barley products, malt and beer. Several studies confirmed its presence in animal-derived food products such as eggs and bovine milk [2].

The potential of DON to act as an endocrine disruptor has been the subject of recent research and continues to be investigated [3]. It has been shown that DON causes a dose-dependent effect on steroid hormones production in animal granulosa cells [3]. The effects of DON on the male reproductive system were studied in mice; this research indicated that serum testosterone concentration was decreased in a dose-related manner, although the exact mechanism of DON action was not identified [4]. Another study evaluated the impact of DON on steroidogenesis of follicle granulosa cells and showed that DON inhibited progesterone (P4) and estradiol (E2) production [5]. It was also observed that DON is able to significantly alter expression of steroidogenesis-related genes in human adrenocortical carcinoma cells, H295R, that express both androgen and estrogen receptors [3]. Based on the standards of the International Agency for Research on Cancer (IARC), DON is classified as compound of Group 3 (inadequate evidence for cancerogenicity in humans) [6]. However, the research data indicate that DON may trigger toxicity in human cells in response to both acute and chronic exposure, especially in the case of low doses [7]. Our previous study showed that DON affects oxidative stress and apoptosis in prostate cancer (PCa) cells in a manner dependent on its hormonal sensitivity [8].

PCa is the most frequently diagnosed cancer in men and second leading cause of male cancer death in the Western world [9]. The majority of patients with newly diagnosed PCa are at low risk for disease progression [10]. Primarily, the development and progression of PCa depends on androgens. The first line of treatment for men with advanced metastatic PCa is androgen deprivation therapy (ADT). As ADT is not curative, PCa often progresses to castration-resistant prostate cancer (CRPC) [11]. Numerous studies confirmed incomplete suppression of prostate tissue androgens as a result of castration therapy. The presence of residual androgens has been also identified in locally recurrent and metastatic castration resistant tumours [12]. Interestingly, the level of testosterone in locally recurrent castrate patients was on a similar or equivalent level as in patients with benign prostatic hyperplasia (BPH); the level of dihydrotestosterone (DHT) was reduced in only 80%—to 0.4 ng/g [13]. Achieving castrate levels of circulating testosterone does not exclude the androgens from the prostate tumour microenvironment produced during so called intertumoral steroidogenesis [14].

Intertumoral steroidogenesis is considered one of the key mechanisms underlying CRPC as a consequence of the reactivation of AR signalling [15]. It has been postulated that testosterone and DHT produced as the result of adrenal androgen conversion constitutes a main source of androgens in PCa after ADT [16,17]. It was reported that PCa cells present an increased level of steroidogenic enzymes that synthesize androgens from cholesterol or other circulating steroid precursors, such as progesterone or DHEA. Intertumoral steroidogenesis has been described as the mechanism that leads to resistance in cases where AR is relaunched in spite of low levels of circulating testosterone [18].

**Figure 1:**
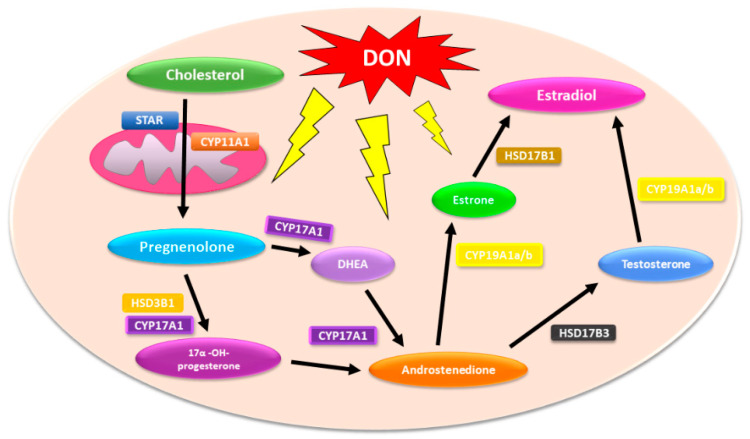
Schematic representation of possible hormone disruptive effects of DON in the human steroidogenic pathway. CYP11A1—Cytochrome P450 Family 11 Subfamily A Member 1; CYP17A1—Cytochrome P450 Family 17 Subfamily A Member 1; CYP19A1 a/b—Cytochrome P450 Family 19 Subfamily A Member 1 a/b; HSD3B1—3 Beta- And Steroid Delta- Isomerase 1; HSD17B3—Hydroxysteroid 17-Beta Dehydrogenase 3; StAR—Steroidogenic acute regulatory protein.

Due to the fact that steroidogenesis is a multistep process that involves the conversion of cholesterol to a comprehensive array of downstream steroids (including testosterone and DHT, via multiple steroidogenic enzymes), environmental agents that affect this process might participate in PCa incidence and progression [19]. On the basis of this observation, it is also possible that DON might affect steroidogenesis in both normal and PCa cells and participate indirectly in cancer progression or resistance (Figure 1).

Previous research indicates that DON affects production of estradiol, testosterone and progesterone in vitro in pig granulosa cells and human adrenocortical carcinoma cells as well as in vivo in rats [2]. However, the effects of DON on steroidogenesis in both normal and cancer prostate cells has not been evaluated yet. Thus, the objective of this study was to determine the effect of DON alone, as well as DON in combination with known the steroidogenic agent DHEA, on the production of estradiol, testosterone and progesterone in prostate normal epithelial cells as well as adenocarcinoma cells with different reported sensitivities to androgens.

## 2. Results

### 2.1. Effects of DON and Co-Treatment with DON and DHEA on Prostate Cell Viability

**Figure 2:**
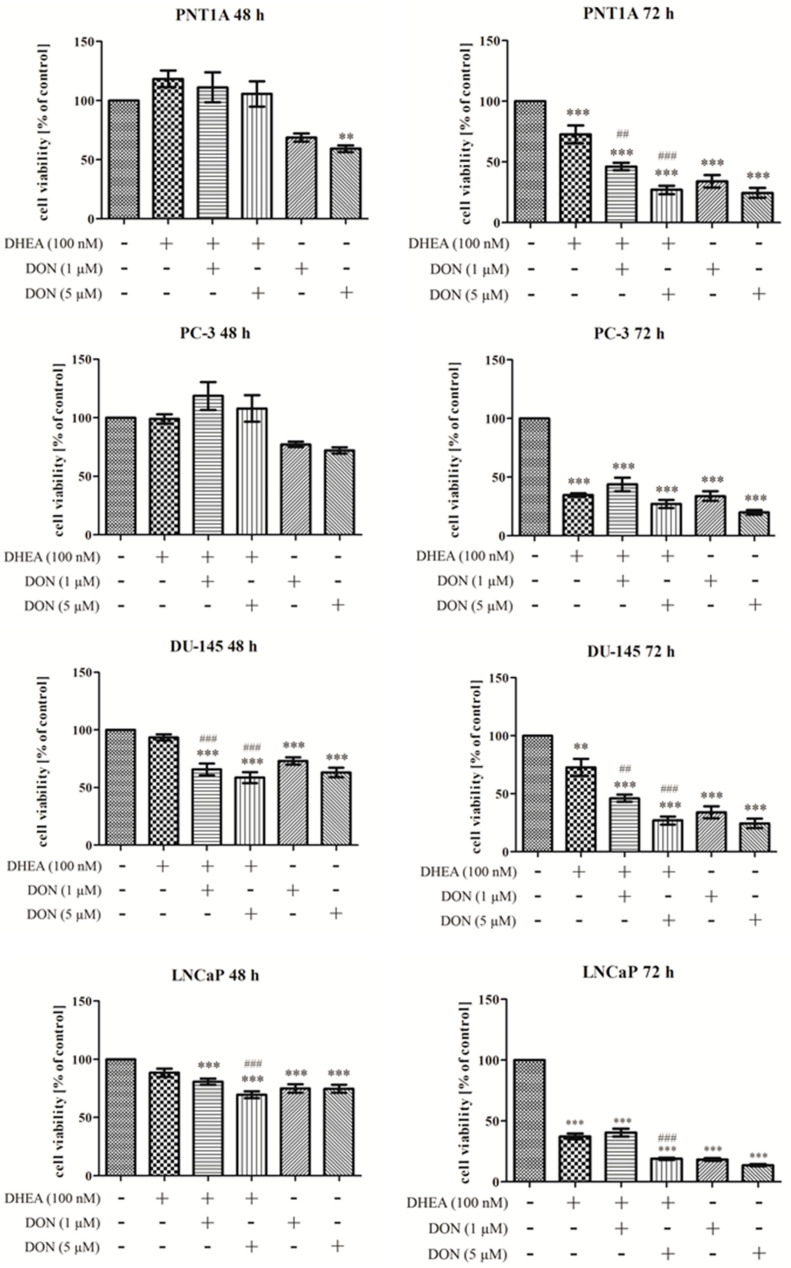
The effects of DON and DHEA treatment on the viability of prostate normal cell line (PNT1A) and cancer cell lines (PC-3, DU-145, LNCaP). The results were obtained by MTT assay and are expressed as the percentage of control cells. The results are expressed as ± SE. *p* < 0.05 was considered statistically significant, *** *p* < 0.001, ** *p* < 0.01, * *p* < 0.05 as compared to control, ### *p* < 0.001, ## *p* < 0.01, # *p* < 0.05, # *p* < 0.05 was considered statistically significant as compared to DHEA (positive control). DON—deoxynivalenol, DHEA—dehydroepiandrosterone, Cnt—control.

Firstly, the viability of cells was evaluated for the chosen doses of DON and combinatory treatment. The doses of DON were based on our previous study [8]; the dose of DHEA was based on the literature survey [20]. After 48 h, we did not observe significant changes in PNT1A cell viability after treatment with 100 nM DHEA, nor after co-treatment with 100 nM DHEA and 1 µM/5 µM DON. Decrease in viability was observed in the case of separate DON treatment, significant for higher doses of DON, but not reaching 50% (Figure 2). After 72 h, 100 nM DHEA caused a significant decrease in PNT1A cell viability, and the dose-dependent effect was also observed after co-treatment with DON (1 µM and 5 µM DON) (*** *p* < 0.001). The dose-dependent effect of DON was also recorded in both used doses. In the case of co-treatment as well as DON treatment alone the viability of the cells did not exceed 50%.

In case of the androgen-independent prostate cancer cell line PC-3, we observed that DHEA (100 nM) did not affect the viability of cells after 48 h exposure. A significant decrease in PC-3 cells viability was observed after 72 h of both separate and combinatory treatments with DON and DHEA. In the androgen-independent DU-145 cells, a decrease in the viability of cells after DON and DON + DHEA treatment was observed after 48 h and was statistically significant, but not reaching the 50% decrease which was noticed after 72 h treatment. In the androgen-dependent cell line LNCaP, a decrease in cell viability was observed after treatment with 1 µM or 5 µM DON or co-treatment with 100 nM DHEA after 48 h compared to control (*** *p* < 0.001), however reduction to 50% was not reached, indicating that androgen-dependent PCa cells are the most sensitive to DON and DON + DHEA treatment. For 72 h exposition, we noted that the decrease in cell viability was similar to the other cell lines tested in the research.

Based on the cell viability experiment, we decided to use the combinatory treatment of 5 µM DON and 100 nM DHEA and 48 h of exposure for the rest of experiments. Although a decrease in cell viability was found for LNCaP cells, it did not exceed 50%, thus this cell line was also used.

### 2.2. Effects of DON and Co-Treatment with DON and DHEA on Hormone Synthesis

**Figure 3:**
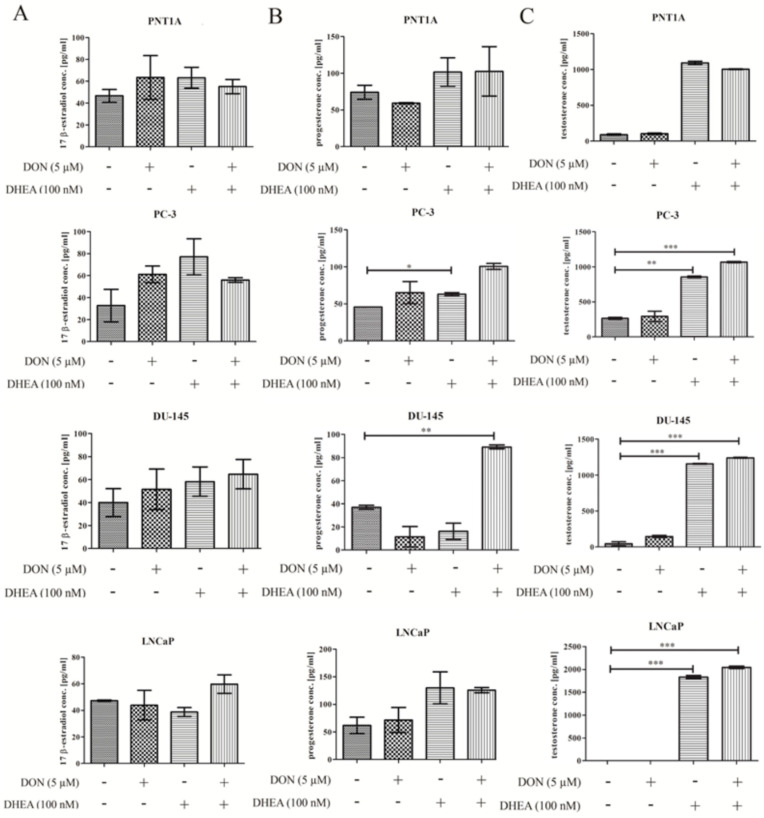
DON and DON and DHEA co-treatment modulates the secretion of steroid hormones after 48 h exposure: 17-β-estradiol (**A**), progesterone (**B**), testosterone (**C**). The results are expressed as ± SE. *p* < 0.05 was considered statistically significant, *** *p* < 0.001, ** *p* < 0.01, * *p* < 0.05 as compared to control. DON—deoxynivalenol, DHEA—dehydroepiandrosterone, Cnt—control.

Next, the influence of DON and DON + DHEA treatment on the production of hormones was evaluated. We observed that 5 µM DON or/and 100 nM DHEA treatment increased the production of 17-β-estradiol in normal prostate epithelial cells (Figure 3A). An elevated expression of 17-β-estradiol was also observed in PC-3 cells treated with DON, DHEA and their combination. A similar tendency was observed in the case of DU-145 cell line: 5 μM DON, 100 nM DHEA and co-treatment of 5 μM DON and 100 nM DHEA resulted in elevated levels of 17-β-estradiol as compared to non-treated cells. For the androgen-dependent cell line LNCaP treatment with 5 µM DON did not affect the level of 17-β-estradiol, and exposure to 100 nM DHEA led to a similar effect. The co-exposure of 5 μM DON and 100 nM DHEA caused an increase in detected 17-β-estradiol in conditioned medium.

Furthermore, the production of progesterone was evaluated. We observed the highest increase in the secretion of progesterone in the cases of the PNT1A and androgen-independent cell lines after co-exposure of DON and DHEA. An increased secretion of progesterone was triggered by DHEA itself in PNT1A cells. In the case of PC-3 cells, both DON and DHEA alone caused increased secretion of progesterone, but to a lower extent than co-treatment. Similar effects were observed in androgen-dependent LNCaP cells.

Our results also indicated that 5 µM DON alone or in combination with 100 nM DHEA induce changes in testosterone production in all cell lines used in the experiment (Figure 3C). In the case of PC-3 cells, treatment with 5 µM DON did not change the level of testosterone as compared to control. The increased level of testosterone was observed after treatment with 100 nM DHEA. The most noticeable changes were detected after co-treatment with 5 μM DON and 100 nM DHEA. Similar to PC-3 cells, exposure of the DU-145 cell line to 5 μM DON did not induce changes in hormone secretion. In case of PNT1A cells this effect was very close to control. The testosterone level in the LNCaP cell line for the control probe was below the detection level. The same was observed after treatment with 5 µM DON. However, we found significant changes after 5 µM DON and 100 nM DHEA co-treatment. In the cases of all tumorigenic cell lines, we observed an increase in the production of testosterone. For the normal prostate cell line PNT1A, the co-treatment caused an increase in production, but it was lower compared to treatment with 100 nM DHEA.

### 2.3. Effects of DON and Co-Treatment with DON and DHEA on the Expression of the Genes and Proteins Related to Steroidogenesis

Subsequently, we evaluated the expression of hormones and steroidogenesis-related genes. Androgen receptor (*AR*) mRNA expression was increased after DON treatment in prostate cancer cell lines PC-3, DU-145 and LNCaP. A decrease was observed for PNT1A cell line as compared to the control. After treatment with 100 nM DHEA, the mRNA expression of AR was slightly elevated or without significant changes in the androgen-independent cell lines PC-3 and DU-145 (Table 1). In case of PNT1A cells, a decrease in relative expression (* *p* < 0.05) of *AR* was observed. As expected for androgen-dependent LNCaP cells, treatment with DHEA increased the mRNA expression of AR. We observed a significant increase of expression of *AR* after co-treatment with 5 µM DON and 100 nM DHEA in PC-3 (** *p* < 0.01) and DU-145 (* *p* < 0.05). However, for PNT1A the effect was opposite, and compared to control the expression was significantly decreased (** *p* < 0.01). For LNCaP cells, co-treatment with DON + DHEA only slightly increased the expression of *AR*.

Treatment of cells with 5 µM DON caused an increase in the expression of estrogen receptor 2 (*ESR2*) in PC-3, DU-145 and LNCaP cells, whereas a contrary effect was observed for PNT1A cells. In all evaluated prostate cancer cell lines, we found a significant increase of expression of *ESR2* (* *p* < 0.05, ** *p* < 0.01) after co-exposure to 5 µM DON and 100 nM DHEA, while for the PNT1A cells the effect was opposite and the expression was significantly decreased (*** *p* < 0.001). In the cases of PNT1A, DU-145 and LNCaP cells, 100 nM DHEA significantly decreased *ERS2* expression.

Only the PC-3 cell line was evaluated for Steroidogenic Acute Regulatory Protein (*StAR*) relative expression (Table 1). 5 µM DON, 100 nM DHEA, and the co-treatment resulted in decreased expression of *StAR*. In the case of PC-3 cell line, the relative expression of StAR protein was decreased after 5 µM DON treatment. 100 nM DHEA and co-treatment of 5 µM DON and 100 nM DHEA caused an increase in relative protein expression (Table 2). In the case of DU-145 and LNCaP cells the expression of StAR was decreased after all experimental treatments, as compared to control. In case of PNT1A cells, the opposite effect was observed.

After exposure to 5 µM DON, we observed that relative expression of Cytochrome P450 Family 11 Subfamily A Member 1 (*CYP11A1*) was elevated comparing to control for PNT1A and PC-3 cells (Table 1). After exposure to 100 nM DHEA, and to the co-treatment of 5 µM and 100 nM DHEA, there was also an increase in the expression of *CYP11A1*. For prostate cancer cell line PC-3 a decrease in expression after DON treatment alone was found. 100 nM DHEA did not affect expression, while co-treatment with 5 µM DON and 100 nM DHEA caused a substantial decrease (ca. two times less compared to control). In the case of LNCaP cell line 5, µM DON treatment did not affect the relative expression of *CYP11A1*; however, we noticed that 100 nM DHEA elevated its expression. A similar effect was observed for the DU-145 cell line. In PNT1A cells we observed that treatment with 5 µM DON did not affect the relative expression of CYP11A1 protein (Table 2). 100 nM DHEA treatment caused an increase, and the effect was similar for co-treatment with 5 µM DON and 100 nM DHEA. For prostate cancer cell lines PC-3, LNCaP and DU-145 it was observed that DON treatment induced an increase in CYP11A1 protein expression. A similar effect was found after 100 nM DHEA and co-treatment with 5 µM DON and 100 nM DHEA, with the exception of cell line DU-145 where, compared to control, the relative expression was decreased for both experimental models.

In normal prostate epithelial cells, the relative expression of Cytochrome P450 Family 17 Subfamily A Member 1 (*CYP17A1*) was not affected after 5 µM DON treatment. Compared to control, there was an elevated level of expression in the case of 100 nM DHEA (* *p* < 0.05) and of co-treatment with 5 µM DON and 100 nM DHEA. In the case of the PC-3 cell line, co-treatment with 5 µM DON and 100 nM DHEA induced the expression of *CYP17A1*, which was not observed in control nor after DON and DHEA treatment alone. In the case of DU-145 there was no increase of expression after the 5 µM DON treatment. We observed a decrease after 100 nM DHEA exposure, while co-treatment with 5 µM DON and 100 nM DHEA caused elevated levels of *CYP17A1* mRNA. For LNCaP, 5 µM DON did not cause changes in expression. There was an elevated level of expression after 100 nM DHEA, but co-treatment decreased the level of *CYP17A1* expression.

In the case of PNT1A and DU-145, DON did not affect the expression of Cytochrome P450 Family 19 Subfamily A Member 1 (*CYP19A1*); however, expression was decreased for the PC-3 and LNCaP cell lines. Treatment with DHEA caused an increase in the expression of *CYP19A1* in of PNT1A and PC-3 cells, whereas a contrary effect was observed for the other tested cell lines. Co-treatment increased the expression of *CYP19A1* in the case of PC-3 cells, but not in normal prostate epithelial cells. Moreover, an opposite tendency was observed for DU-145 and LNCaP cells—a reduced expression pattern was found.

The relative expression of Hydroxy-Delta-5-Steroid Dehydrogenase, 3 Beta- And Steroid Delta-Isomerase 2 (*HSD3B2*) was not affected by DON treatment in the PNT1A cell line, but increased after DHEA and DON+DHEA treatment. For the PC-3 cell line we observed relatively low expression of *HSD3B2* after treatment with DON and DHEA, but it increased significantly (* *p* < 0.05) after co-treatment with 5 µM DON and 100 nM DHEA. In the LNCaP cell line the relative expression in the control was at a low level. Treatment with 5 µM DON induced expression. A similar effect was observed with 100 nM DHEA treatment, but the increase was ca. 10 times greater compared with control. In DU-145 cells the experimental treatment of 5 µM DON or 100 nM DHEA resulted in the increase of expression of *HSD3B2*. There was no change in expression after co-treatment with 5 µM DON and 100 nM DHEA.

The expression of Hydroxysteroid 17-Beta Dehydrogenase 2 (*HSD17B2*) was investigated only in the case of the PC-3 cell line; 100 nM DHEA did not influence its levels. Treatment with 5 µM DON and co-treatment with 5 µM DON and 100 nM DHEA both resulted in a significant increase of *HSD17B2* expression (* *p* < 0.05).

**Figure 4:**
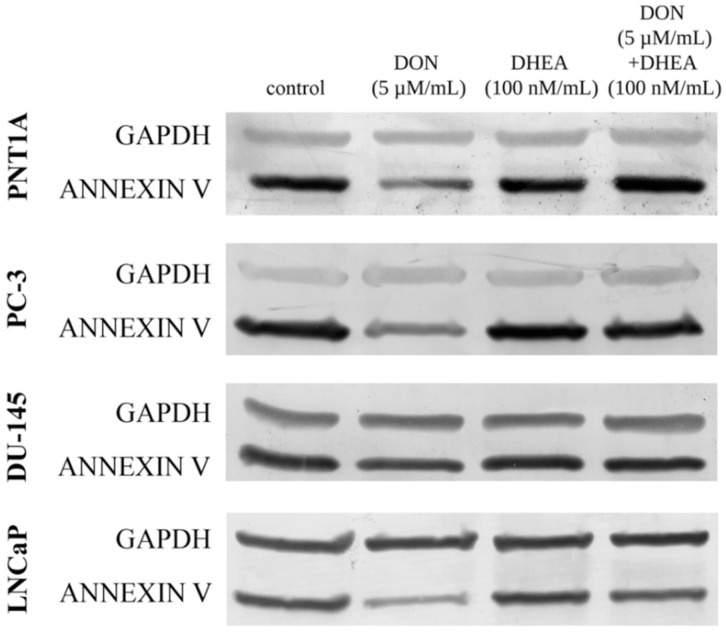
The representative results of Western Blot analysis for annexin V protein. DON—deoxynivalenol; DHEA—dehydroepiandrosterone.

We also observed a modulation of the expression of annexin A5 (*ANX5A*) in both normal as well as in cancer cell lines (Table 3). In normal prostate epithelial cells, DON and DHEA treatments alone caused an increase in *ANX5A* expression, but the combinatory treatment showed a lower increase as compared to separated treatments. In the case of all tested PCa cells, DON itself and DON + DHEA treatment caused a significant increase in the expression of *ANX5A*. In LNCaP and DU-145 cells, an increase was also observed after DHEA treatment alone. No such effect was detected in PC-3 cells. Although significant changes in *ANX5A* expression were observed at the level of genes, no such effect was observed at the level of proteins. A detectable decrease was observed only for DON treatment, both in the normal and in the cancer cell lines (Figure 4). A co-treatment of 5 µM DON and 100 nM DHEA elevated the expression of *ANX5A* in the cases of PNT1A, PC-3 and DU-145, however the changes were very low.

### 2.4. Effects of DON and Co-Treatment with DON and DHEA on Apoptosis and Cell Cycle Progression

**Figure 5:**
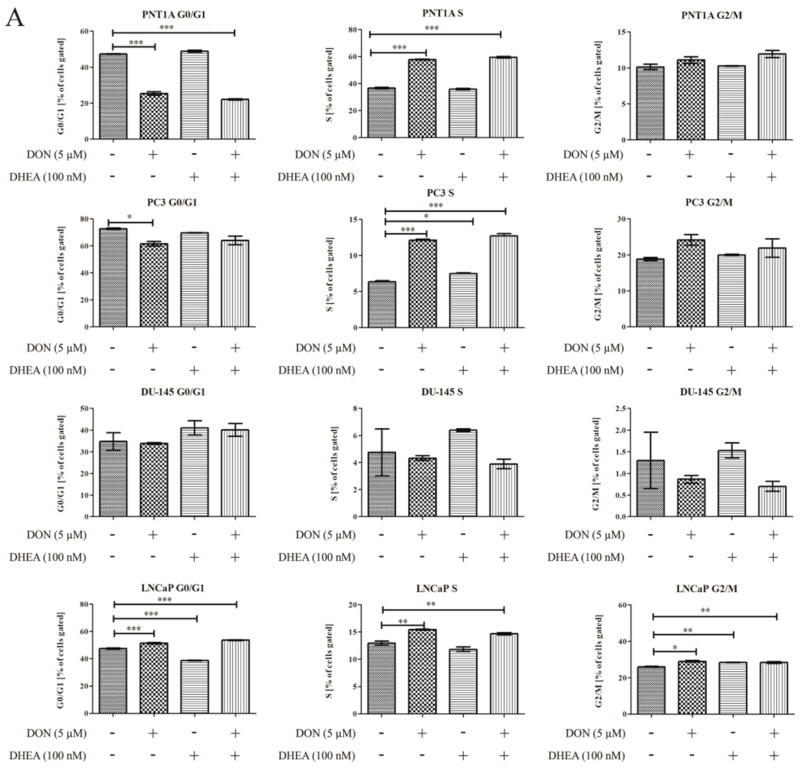

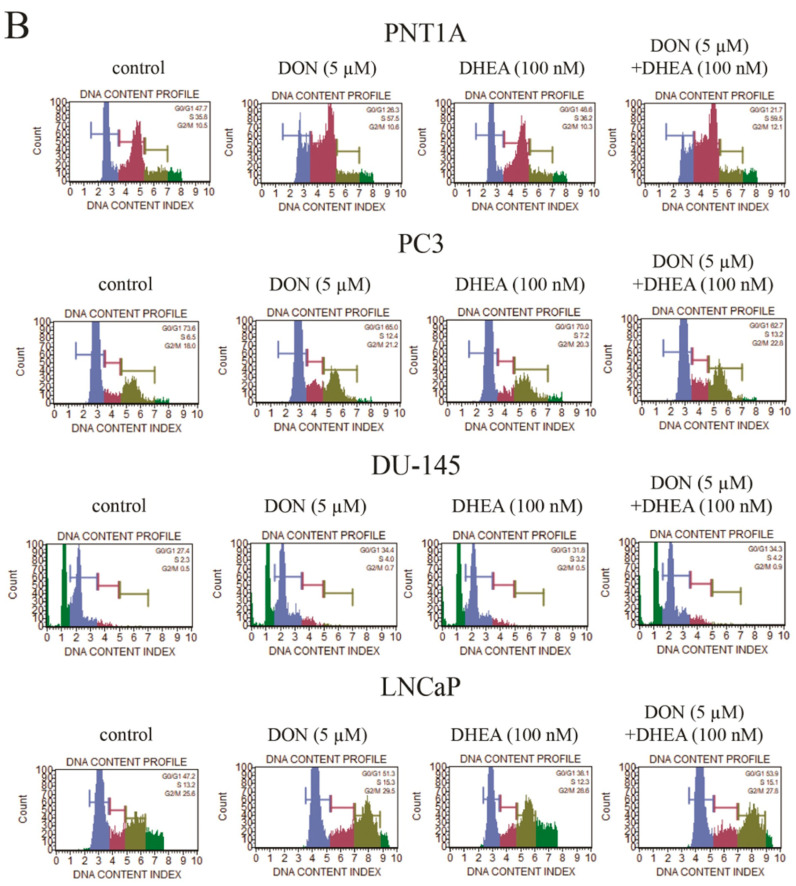
The influence of DON, DHEA, and a co-treatment of DON and DHEA on cell cycle progression after 48 h exposure. The number of PNT1A, PC-3, DU-145, LNCaP cells in G0/G1, S, G2/M, respectively, is expressed as the percentage of gated cells (**A**); representative results obtained by flow cytometry with the Cell Cycle Analysis Kit (**B**). Results are expressed as ± SE. *p* < 0.05 was considered as statistically significant, *** *p* < 0.001, ** *p* < 0.01, * *p* < 0.05 as compared to control. DON—deoxynivalenol; DHEA—dehydroepiandrosterone.

Firstly, the possible effects of DON and DON+ DHEA treatment on cell cycle progression were evaluated (Figure 5). In the case of PNT1A we observed that after treatment with 5 µM DON and co-treatment with 5 µM DON and 100 nM DHEA the number of cells gated in the G0/G1 phase was significantly decreased (*** *p* < 0.001). 100 nM DHEA did not cause a change in the number of cells gated in the G0/G1 phase, compared to control. We observed a significantly higher number of cells gated in the S phase after treatment with 5 µM DON and with 5 µM DON and 100 nM DHEA (*** *p* < 0.001). Treatment with 100 nM DHEA did not affect the cells in the S phase. The number of cells gated in G2/M was also increased after 5 µM DON treatment and a co-treatment of 5 µM DON and 100 nM DHEA; however, this effect was not significant.

In the case of the androgen-independent PC-3 and DU-145 cell lines, treatment with DON caused the decrease of the number of cells gated in the G0/G1 phase. For the PC-3 cell line the effect was significant (* *p* < 0.05). After co-treatment with 5 µM DON and 100 nM DHEA the number of gated cells was also decreased, but not in a significant manner. No change was observed after treatment with 100 nM DHEA. There were some changes observed in the S phase. The number of cells gated was significantly increased in the PC-3 cell line (*** *p* < 0.001) after 5 µM DON treatment and 5 µM DON and 100 nM DHEA co-treatment. In the case of DU-145 in the S phase there were fewer cells gated, compared to control, after both 5 µM DON treatment and 5 µM DON/100 nM DHEA co-treatment. In the G2/M phase we observed that 5 µM DON and 5 µM DON/100 nM DHEA co-treatment caused an increase in gated cells. The effect was reversed in the DU-145 cell line, where we observed a decrease in the number of gated cells in the S phase. In both cell lines, treatment with 100 nM DHEA did not affect cell cycle progression. In the androgen-dependent LNCaP cell line, the number of cells gated in the G0/G1, S and G2/M phases increased significantly; the same effect was found after co-treatment with 5 µM DON and 100 nM DHEA (*** *p* < 0.001; ** *p* < 0.01). Treatment with 100 nM DHEA did not result in changes in the number of gated cells compared to control.

**Figure 6:**
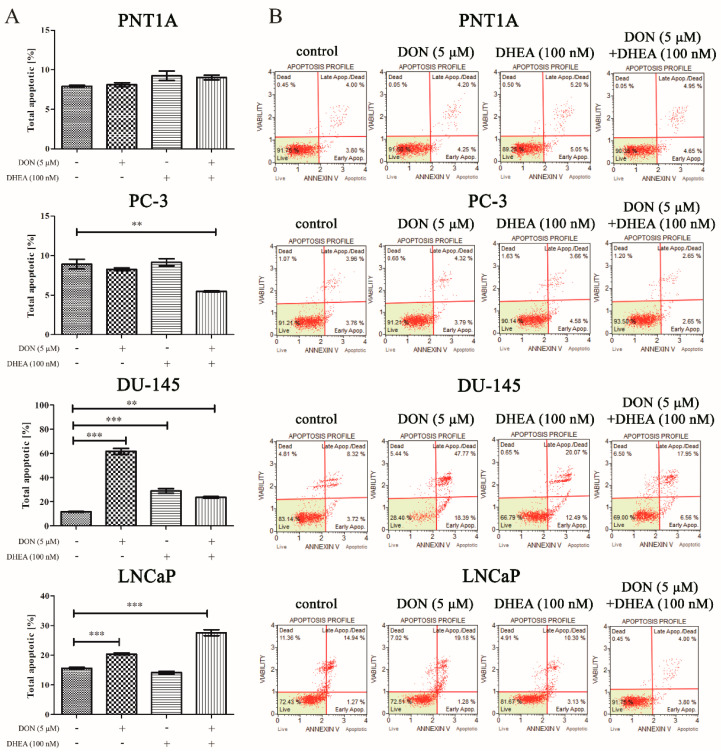
The influence of DON, DHEA, and a co-treatment of DON and DHEA on apoptosis Indictable 48 h exposure: the number of PNT1A, PC-3, DU-145, LNCaP gated cells (early and late apoptotic) is presented as the percentage of gated cells (**A**); representative results of the apoptosis profile obtained by flow cytometry with the Apoptosis Assay for Muse Cell Analyzer (**B**). Results are expressed as ± SE. *p* < 0.05 was considered as statistically significant, *** *p* < 0.001, ** *p* < 0.01, * *p* < 0.05 as compared to control. DON—deoxynivalenol, DHEA—dehydroepiandrosterone.

As the next step, we evaluated the effect of DON, DHEA, and co-treatment with DON and DHEA on programmed cell death, or apoptosis (Figure 6). Not significant and not remarkable increase in the number of apoptotic cells was observed in normal prostate epithelial cells in all tested doses of DON and DHEA. In the androgen-independent cancer cell line, PC-3, neither treatment with DON nor with DHEA caused changes in the induction of apoptosis, while a decreased number of apoptotic cells was observed with co-treatment (** *p* < 0.01). In another androgen-independent cell line, DU-145, we observed an increased number of apoptotic cells after treatment with DON (** *p* < 0.01); compared to control, the number of the apoptotic cells was four times higher. Treatment with DHEA and co-treatment with DON and DHEA also caused an increase in apoptotic cells compared to the control (*** *p* < 0.001; ** *p* < 0.01). We also analysed the androgen-dependent prostate cancer cell line LNCaP; Treatment with DON and co-treatment with DON and DHEA both caused an increase in the number of total apoptotic cells (** *p* < 0.01) compared to control. This effect was not present for DHEA, where we did not observe an elevated level of apoptotic cells.

## 3. Discussion

It is believed that the hormonal balance plays a crucial role in PCa incidence [21]. The switch in the androgen/estrogen ratio which naturally occurs with age and the ability of prostate cells to synthetize androgens de novo, confirm this assumption. Thus, intratumoral steroidogenesis might have a great impact on tumor progression. DON was previously reported to compromise productivity in pigs (i.e., impaired oocyte and embryo development, decreased body weight and sperm quality in male pigs) and reduce feed intake in animals [22]. Steroid hormone concentration imbalance can be linked to the effects of impaired fertility and modulated secretion of progesterone from granulosa cells caused by DON exposure [5]. Our previous study showed that DON affects oxidative stress and apoptosis in prostate cancer cells, however the effect is dependent on the androgen-sensitivity of cells [8]. A study conducted by Liu et. al. showed that DHEA can decrease the cell proliferation of primary Leydig cells in a dose-dependent manner; the cell viability may be improved in a time-dependent and dose-dependent manner [23]. It has been reported that DHEA inhibits the proliferation of several types of cancer cells, including hepatoma and myeloma cancer cells [24,25]. DHEA can act as an inducer of the proliferation of estrogen and androgen receptor-positive breast cancer cells, and inhibit the proliferation of estrogen receptor-negative cells [26,27]. Thus, in this study, we decided to evaluate the effect of DON and DON+DHEA treatment on steroidogenesis in prostate normal and cancer cell lines.

To evaluate the ability of the human prostate to synthesize steroids de novo, we examined the expression of key enzymes and proteins involved in steroid biosynthesis and metabolism [28]. The conversion of testosterone to estradiol is mediated by the enzyme aromatase, which is highly expressed in fat tissue and might also be present in the prostate [29]. This raises the possibility that prostate cancer is in part induced by testosterone due to estrogen effects [30,31]. Our results indicated that treatment with 5 µM DON caused an elevated level of testosterone production compared to control. We observed this effect in androgen-independent PC-3 and DU-145 cells. In PNT1A cells the effect was very near control. We observed an increase in the production of testosterone in the case of all tumorigenic cell lines. To the best of our knowledge, this is the first study reporting that DON and DON+DHEA treatment modulates the production of testosterone in human PCa cells. Interestingly this observation is contrary to a previous one where DON strongly inhibited testosterone production in MA-19 Leydig cells after 48 h exposure at concentrations of 8–16 µM [32]. Another group also demonstrated that high-dose exposure to DON (1000 ng/mL) inhibited the release of testosterone in bovine ovarian cells [31]. A study conducted by Sprando et al. on male rats showed that testosterone concentrations were decreased in a dose-related manner across all dose groups [3]. This difference might be due to possible differences between intratumoral steroidogenesis and steroidogenesis taking place in normal cells.

In our study, treatment with DON caused increased progesterone concentration compared to control in both PC-3 and LNCaP. The opposite effect was observed in DU-145 and PNT1A cells, where the level of progesterone was lower than in control. A similar tendency was noted when the cells were treated with DHEA. However, the results indicate that for all cell lines the observed effect was stimulatory with respect to co-treatment with DON and DHEA. As showed by Kolesarova et al., DON can influence the synthesis and upregulate the level of progesterone in porcine granulosa cells isolated from the ovary in high doses (1000–5000 ng/mL), with low doses not affecting secretion [33]. In contrast, another study showed that a high dose of DON (1000 ng/mL) inhibited progesterone production [34], and a similar effect was observed with a combination of DON and fumonisin B1 [35]. On the other hand, in the study conducted by Guerrero-Netro et al., DON in low doses (0–100 ng/mL) had an inhibitory effect on progesterone secretion in porcine granulosa cells [27], whereas in bovine theca cells low doses stimulated the release of this hormone [36]. In bovine ovarian cells low doses (10 ng/mL) promoted progesterone secretion, while high doses (1000 ng/mL) reduced it [31]. Pizzo et al. reported an inhibitory effect on progesterone secretion in small-follicle bovine granulosa cells and cattle granulosa cells in high doses (1000 ng/mL) [5,37]. In studies performed on the MA-10 Leydig cell line, DON was shown to promote progesterone secretion in higher doses (8–16 µM) [37], whereas a low dose (0.1 µM) reduced the hormone release [38].

As presented by other groups, estradiol production can also be inhibited by DON in a dose-dependent manner. Either high or low doses can disturb estradiol production by decreasing the level of the hormone in porcine and bovine granulosa cells [5,27,35,37]. However, Kolesarova et al. showed that high doses of DON (2000–5000 ng/mL) stimulated estradiol production [33]. Additionally, other groups showed that even low doses can act to promote estradiol release [34]. In our study, DON (5 µM) promoted estradiol production both alone and in combination with DHEA in a dose- and cell type-dependent manner.

The process of steroidogenesis involves several enzymes that catalyse the production of steroids, which can be disturbed by DON through interference with the 60S ribosomal subunit and termination of translation [39,40]. To determine the effect of DON on steroidogenesis we evaluated the expression of genes related to steroidogenesis– *CYP11A1, CYP17A1, CYP19A1, HSD3B2, HSD17B2* and *StAR* using RT-qPCR. We were also interested in *ANX5A*, due to the fact that annexin V is considered to regulate testosterone production [41]. We observed the highest relative expression of *CYP11A1* in the PC-3 line compared to the rest of the investigated cell lines, and the differences were noticeable. There were no significant differences after the addition of 100 nM DHEA. However, in the presence of DON we observed that the combined effect of DON and DHEA caused a decrease in the expression of *CYP11A1*. The inhibitory effect was even stronger after exposure to 5 µM DON. Interestingly, in our study the PC-3 cell line was the only one where we were able to assess *StAR* expression at a detectable level. Relative expression of *StAR* after exposure to DON was decreased at a very similar level as co-treatment with 100 nM DHEA. We observed that the smallest changes in mRNA expression were present in the androgen-dependent LNCaP cell line; however, remarkable changes were observed on the protein level. This indicates that in androgen-dependent cells DON and DHEA affect protein synthesis, but not gene expression. In the study conducted by Guerrero–Netro et al., DON was reported not to alter the *CYP11A1* and *StAR* expression in bovine granulosa cells from follicles, but to potently suppress the CYP19A1 expression [42]. Other studies showed that the expression of *CYP11A1* and *CYP19A1* can be stimulated by exposure to DON (3.3 µM) in small follicle granulosa cells [5].

Following this, we investigated the next enzyme present in the steroidogenic pathway, CYP17A1. This enzyme, also called steroid 17α-monooxygenase, catalyses many reactions and is considered to be the key enzyme in the pathway. In our study, the expression of *CYP17A1* was identified in all investigated cell lines and the highest increase in expression was observed for co-treatment of DON and DHEA. An up-regulation of the expression of *CYP17* was also observed in human adrenal gland carcinoma cell lines [3]. Next in our scope of interest was Hydroxy-Delta-5-Steroid Dehydrogenase, an enzyme responsible for conversion of pregnenolone to progesterone that is encoded by *HSD3B2*. When we observed the differences in the rate of relative expression, the highest was for the LNCaP cell line, and the lowest for PNT1A. In a study conducted by Ndossi et al., DON (100 ng/mL) was found to upregulate the expression of *HSD3B2* in the H295R human adrenocortical carcinoma cell line [3]. Additionally, we investigated the gene *HSD17B2* that encodes 17β-Hydroxysteroid Dehydrogenase (17β-HSD). In our study we identified detectable level of the gene in the PC-3 cell line in the RT-qPCR experiment. As reported by Gao et al., there are multiple mechanisms of *HSD17B2* regulation in prostate cancer [40]. It was confirmed that the highest expression was detected in the PC-3 line, while in other cell lines (i.e., DU-145) gene deletion or DNA methylation could be the mechanism of silencing *HSD317B2* expression.

Moreover, we evaluated the influence of DON and DHEA on cell cycle progression in all cell lines used in this study. The results showed that DON caused a decrease of the number of cells in G0/G1 in the investigated cell lines PNT1A, PC-3 and DU-145, but not in LNCaP. This is in accordance with a study performed in our laboratory where the influence of several doses of DON on prostate cancer cell lines was investigated [8]. DHEA did not cause any significant changes in the number of cells in the G0/G1 phase compared to control. However, co-exposure to 5 µM DON and 100 nM DHEA in case of the PNT1A cell line significantly decreased the number of gated cells. As showed by Diesing et al., DON affects the cell cycle progression of the Caco-2 cell line in doses of 2000 ng/mL. Cell exposure resulted in a significant decrease in the G0/G1 phase after 48 and 72 h. There was also a numerical increase observed in cells in the G2/M phase after 72 h DON treatment [43]. In our study, DON was found to increase the number of cells in the G2/M phase for the PNT1A and PC-3 cell lines. Similar observations were demonstrated in other studies on HepG2 cells, where DON was also found to cause cell cycle arrest in the G0/G1 and G2/M phases [42,44]. We observed that in the case of DU-145 the result is reversed; the percentage of G2/M cells was decreased comparing to control. There were no significant changes in the number of G2/M cells after treatment with 100 nM DHEA. The co-treatment of 100 nM DHEA and 5 µM DON caused an increase in the percentage of G2/M cells. However, the result was reversed for the DU-145 cell line. We also demonstrated that apoptosis is induced after treatment with DON, which is in accordance with our previous study [8], whereas DHEA did not promote apoptosis. However, co-treatment of DON and DHEA caused an increase in the percentage of apoptotic cells in the normal human prostate cell line, PNT1A, and the androgen-dependent prostate cancer cell line LNCaP.

To the best of our knowledge, this is the first study to show that DON might affect local steroidogenesis in normal prostate epithelial cells and PCa cells, especially in combination with other hormones like DHEA. Taken together, the results of this study suggest that DON is able to influence the various steps of the steroidogenesis process in normal prostate and prostate cancer cell lines in vitro. Moreover, our findings indicate that co-exposure to mycotoxin and hormone active food supplements affect cell cycle progression and activation of apoptosis. The molecular implications underlying the modulation of steroidogenesis by DON need to be further investigated.

## 4. Materials and Methods

### 4.1. Cell Lines and Experimental Protocol

Human prostate adenocarcinoma cell lines LNCaP, PC-3 and immortalized normal prostate epithelial PNT1A cells were obtained from the European Collection of Authenticated Cell Cultures (ECACC) (Sigma-Aldrich, Saint Louis, MO, USA), whereas the DU-145 cells were obtained from the American Type Culture Collection (ATCC).

The cells were routinely maintained in RMPI (LNCaP, PC-3 and PNT1A) or DMEM (DU-145) with 10% heat inactivated fetal bovine serum (FBS) in a humidified atmosphere of 5% CO_2_ at 37 °C. Growth medium was supplemented with 2 mM L-glutamine, 1 mM sodium pyruvate, 10 mM HEPES and 1% of PenStrep (50 Ul/mL and 50 µg/mL streptomycin). All media and supplements were purchased from Thermo Fisher Scientific Inc., Waltham, MA, USA.

### 4.2. Experimental Treatments

To determine the effects of DON on PCa steroidogenesis, cells were treated with 0, 1 or 5 µM DON or DHEA 0–100 nM over 2 or 3 days. Non-treated cells were used as a control. Deoxynivalenol (DON) (Sigma-Aldrich, Saint Louis, MI, USA) and dehydroepiandrosterone (DHEA) (Avanti Polar Lipids, Inc., Alabaster, AL, USA) stock solutions were prepared in ethanol. Stocks were dissolved in experimental medium before use.

The doses and experimental scheme were based on our previous study [8]. All experiments were carried out with three different pools of cells. Because DHEA can serve as a precursor for the more potent androgens T and DHT [44], as well as estrogens, its effects on prostate health are of interest.

### 4.3. Cell Viability Assays

The colorimetric MTT (3-(4,5-dimethylthiazol-2-yl)-2,5-diphenyltetrazolium bromide) (Merck Millipore, Burlington, NJ, USA) assay was used to assess the viability of the cells after treatment with DON and DHEA. The MTT test was performed according to the manufacturer’s instructions in order to determine the concentration of DHEA that does not affects cell viability (more than 80% of viable cells). The cells at concentration 1 × 10^5^ were seeded in 100 µL of culture media to reach ca. 85–90% of confluence. Next the cells were treated with the experimental doses of DHEA (10–100 nM) for 48 h and 72 h. 10 µL MTT solution (5 mg/mL) was added to each well 4 h prior to the incubation ending. Then, the formed formazan crystals were dissolved by adding 100 µL of solvent—10% SDS with 0.01 M HCl for overnight incubation in 37 °C. The absorbance at 570 nm was recorded using and ELX 80IU microplate reader (BioTek, Winooski, VT, USA).

### 4.4. Cell Cycle

Propidium iodide (PI) staining in the presence of RNAse was used to evaluate the percentage of cells in the G0/G1, S and G2/M phases of the cell cycle with the Muse^®^ Cell Cycle Assay Kit (Merck Millipore, Burlington, MA, USA). Cells (3 × 10^5^/well) were seeded on 6-well plates and cultured to approach 90% of confluence. Next, the cells were treated with experimental media containing 2 μM, 3 μM and 5 μM of DON for 24 h and trypsinized. The Cell Cycle Assay was conducted according to manufacturer’s recommendations. Cells were analyzed with the Muse™ Cell Analyzer (Merck Millipore, Burlington, MA, USA). The results were expressed as the percentage of cells in each cell cycle phase. The experiment was conducted in triplicate.

### 4.5. Annexin V and Dead Cell Double Staining Assay

Cell apoptosis was determined with the use of Muse Annexin V and Dead Cell Kit (Merck Millipore, Burlington, MA, USA). The cells (3 × 10^5^/well) were seeded on 6-well plates and cultured to until 90% confluence. After exposure to 5 µM DON, 100 nM DHEA or co-exposure to 5 µM and 100 nM DHEA for 48 h, cells were detached and suspended in 100 µL of culture medium. The assay was performed according to the manufacturer’s instructions. The experiment was conducted in triplicate.

### 4.6. Steroid Assays

The levels of the steroid hormones progesterone, testosterone, 17-β-estradiol were determined via enzyme-linked immunosorbent assay (ELISA). This method is based on competitive colorimetric detection. The cells were seeded in 6-well plates (1 × 10^6^/well) and treated with the experimental doses of DON (5 µM) and DHEA (10–100 nM) for 48 h and 72 h. Progesterone, testosterone, and 17-β-estradiol were measured in conditioned medium collected after 48 h and 72 h experimental treatment. The experiment was performed in duplicate. ELISA assays (Enzo Chemicals Inc., Farmingdale, NY, USA) were performed according to the manufacturer’s instructions. The absorbance was measured at 405 nm. The sensitivity of the assays was 8.57 pg/mL (progesterone), 5.67 pg/mL (testosterone) and 28.5 pg/mL (17-β-estradiol).

### 4.7. RNA Extraction and Real Time Quantitative Polymerase Chain Reaction (RT-qPCR)

For RNA experiments, RNA was collected in triplicate from each cell line. The cells were cultured on 60 mm Petri dishes. After treatment, medium was removed and total RNA was extracted using TRIzol Reagment (Thermo Fisher Scientific Inc., Waltham, MA, USA), next isolated according to manufacturer’s instructions and then diluted in 50 μL of sterile deionized water. The concentration of RNA was measured using the BioDrop DUO spectrophotometer (BioDrop, Cambridge, UK). cDNA (5 μg) was synthetized using ImProm RT-IITM reverse transcriptase (Promega, Madison, WI, USA). Real-time PCR was performed on Light Cycler 96 (Roche, Basel, Switzerland). Target genes were: cytochrome P450 Family 11 Subfamily A Member 1 (*CYP11A1*)*,* cytochrome P450 Family 17 Subfamily A Member 1 (*CYP17A1*)*,* cytochrome P450 Family 19 Subfamily A Member 1 (*CYP19A1*)*,* 3 Beta- And Steroid Delta- Isomerase 2 (*HSD3B2*)*,* Hydroxysteroid 17-Beta Dehydrogenase 2 (*HSD17B2*), steroidogenic acute regulatory protein (*StAR*), annexin 5 (*ANX5A*), estrogen receptor 1 (*ESR1*), estrogen receptor 2 (*ESR2*) and androgen receptor (*AR*). The human reference RNA (Stratagene, San Diego, CA, USA) was used as the calibrator. Primers were designed and verified with the Primer BLAST software (National Institute of Health, NIH, Bethesda, MD, USA). The Table 4 presents the sequences of the primers used. The relative expression was normalized to ribosomal protein S17 (*RPS17*), ribosomal protein P0 (*RPLP0*) and histone H3.3A (*H3F3A*) used as a housekeeping gene. In order to avoid detection of non-specific products for each reaction, melting curve analyses was performed to verify the product identity. The obtained data were analyzed using ΔΔCt method.

### 4.8. Western Blot

The cells were seeded on 100 mm Petri dishes at a density of 1 × 10^6^ cells/dish. After experimental treatments with DON and DHEA, total protein extracts were isolated with the use of an RIPA protein extraction buffer (Sigma Aldrich, Saint Louis, MO, USA), supplemented with protease and phosphatase inhibitor cocktails (Sigma-Aldrich, Saint Louis, MO, USA) and 1 mM PMSF (Sigma Aldrich, Saint Louis, MO, USA). Direct Detect^®^ (Merck Millipore, Burlington, MA, USA) was used to determine protein concentration. Equal amounts of protein (30 μg) were used for electrophoresis. Firstly, the samples were mixed with Laemmli Lysis-Buffer and heated at 100 °C for 5 min. Subsequently, samples were resolved on 12.5% SDS-polyacrylamide gels (120 V, 2 h) and then electrophoretically transferred onto PVDF membranes (Merck Millipore, Burlington, MA, USA) by wet blotting (400 mA, 70 min) using Mini Trans-Blot^®^ Cell (Bio Rad, Hercules, CA, USA). We used 0.1% Panceau-S (Sigma Aldrich, Saint Louis, MO, USA) in 1% acetic acid for protein visualization. Next, membranes were blocked in 5% non-fat milk in TBST for 1 h at RT.

For colorimetric detection, they were incubated overnight at 4 °C with primary antibody, according to the manufacturer’s instructions: Annexin V (#8555) (Cell Signaling Technology, Leiden, The Netherlands). Then, the membranes were washed with TBST buffer (3 × 5 min) and incubated with 1% non-fat milk solution (1:15,000) of secondary antibody conjugated with alkaline phosphatase (Sigma Aldrich, Saint Louis, MO, USA) at 4 °C for 4 h. Following this step, membranes were washed with the TBST buffer (3 × 5 min). The bands were visualized with Novex^®^ AP Chromogenic Substrate (Life Technologies, Carlsbad, CA, USA).

For chemiluminescence detection, the membranes were incubated overnight at 4 °C with primary antibodies: STAR (#8449), CYP11A1 (#14217) (Cell Signaling Technology, Leiden, The Netherlands). Next, the membranes were washed with TBST buffer (3 × 15 min) and then incubated with the solution (1: 5000) in 1% non-fat milk in TBST of secondary antibody conjugated with horseradish peroxidase (HRP) (Thermo Fisher Scientific Inc., Waltham, MA, USA). The bands were visualized with the use of The ChemiDoc MP Imaging System (Bio Rad, Hercules, CA, USA).

The densitometric analysis of protein levels was performed with the use of Image Lab Software (Bio Rad, Hercules, CA, USA). The results were normalized to GAPDH (sc-059540) (Santa Cruz Biotechnology Inc., Dallas, TX, USA) as the reference protein.

### 4.9. Statistical Analysis

All statistical analyses were performed with the GraphPad Prism Software (Graphpad Software, San Diego, CA, USA). Data was analyzed using one-way ANOVA to find the statistical differences and the results are expressed as ± SE. *p* < 0.05 was considered as statistically significant.

## Figures and Tables

**Table 1:** The relative expression of the genes associated with steroidogenesis. The results are expressed as a mean.

Cell Line	Treatment	*AR*	*ESR2*	*CYP11A1*	*CYP17A1*	*CYP19A1*	*HSD3B2*	*HSD17B2*	*StAR*
PNT1A	cnt	0.25	10.06	0.21	0.7	0.85	0.05	-	-
	5 μM DON	0.17	3.73 **	0.29	0.51	0.71	0.09	-	-
	100 nM DHEA	0.09 *	1.21 ***	0.31	4.3 *	1.62	0.73	-	-
	5 μM DON + 100 nM DHEA	0.05 **	0.20 ***	0.35	2.82	0.85	0.29	-	-
PC-3	cnt	19.78	19.78	2.13	0.08	0.6	0.21	19.24	1.33
	5 μM DON	39.72	39.72	0.15	0.13	0.12	0.21	70.73	0.14
	100 nM DHEA	19.21	19.21	2.27	0.17	0.92	0.15	12.17	0.4
	5 μM DON + 100 nM DHEA	97.83 **	97.83 **	0.85	3.86 **	5.41 *	9.73 ***	284.71 *	0.15
DU-145	cnt	0.08	0.88	0.25	9.44	2.6	1.34	-	-
	5 μM DON	0.17	6.87	0.28	9.66	2.55	3.61	-	-
	100 nM DHEA	0.05	0.62	0.28	6.16	1.29	3.69	-	-
	5 μM DON + 100 nM DHEA	0.26	10.59	0.43	16.8	1.86	1.41	-	-
LNCaP	cnt	0.72	0.66	0.28	8.93	2.18	0.89	-	-
	5 μM DON	0.95	1.05	0.26	115.9	1.15	2.28	-	-
	100 nM DHEA	2.72	0.54	0.37	24.73	0.85	11.45	-	-
	5 μM DON + 100 nM DHEA	0.98	5.92 **	0.21	9.12 **	1.06	4.36	-	-

* p < 0.05 was considered as statistically significant, ** p < 0.01, *** p < 0.001. AR—androgen receptor; ESR2—estrogen receptor 2; CYP11A1—Cytochrome P450 Family 11 Subfamily A Member 1; CYP17A1—Cytochrome P450 Family 17 Subfamily A Member; CYP19A1—Cytochrome P450 Family 19 Subfamily A Member 1; HSD3B2—Hydroxy-Delta-5-Steroid Dehydrogenase, 3 Beta—And Steroid Delta-Isomerase 2; HSD17B2—Hydroxysteroid 17-Beta Dehydrogenase 2; StAR—Steroidogenic Acute Regulatory Protein; DON—deoxynivalenol; DHEA—dehydroepiandrosterone; cnt—control.

**Table 2:** The relative protein expression of the mediators of the initial and rate-limiting step in steroidogenesis—CYP11A1 and StAR. The results are expressed as a mean.

Cell Line	Treatment	CYP11A1	StAR
PNT1A	cnt	1.17	0.07
	5 μM DON	1.04	0.05
	100 nM DHEA	1.62	0.06
	5 μM DON + 100 nM DHEA	2.88	0.08
PC-3	cnt	0.07	0.19
	5 μM DON	0.07	0.16
	100 nM DHEA	0.16	0.25
	5 μM DON + 100 nM DHEA	0.42	0.29
DU-145	cnt	0.32	0.12
	5 μM DON	0.39	0.05
	100 nM DHEA	0.23	0.04
	5 μM DON + 100 nM DHEA	2.24	0.02
LNCaP	cnt	0.36	0.68
	5 μM DON	2.32	0.11
	100 nM DHEA	1.90	0.12
	5 μM DON + 100 nM DHEA	1.85	0.11

CYP11A1—Cytochrome P450 Family 11 Subfamily A Member 1; StAR—Steroidogenic Acute Regulatory Protein; DON—deoxynivalenol; DHEA—dehydroepiandrosterone; cnt—control.

**Table 3:** The relative expression of *ANX5A* analyzed by RT-qPCR. The results are expressed as a mean.

Cell Line	Treatment	*ANX5A*
PNT1A	cnt	0.69
	5 μM DON	1.22
	100 nM DHEA	1.27
	5 μM DON + 100 nM DHEA	0.78
PC-3	cnt	14.83
	5 μM DON	23.32
	100 nM DHEA	14.64
	5 μM DON + 100 nM DHEA	53.69 ***
DU-145	cnt	0.7
	5 μM DON	3.48 *
	100 nM DHEA	1.31
	5 μM DON + 100 nM DHEA	3.95 **
LNCaP	cnt	0.17
	5 μM DON	0.37
	100 nM DHEA	0.34
	5 μM DON + 100 nM DHEA	0.45 *

* p < 0.05 was considered statistically significant, ** p < 0.01, *** *p <* 0.001 as compared to the control. DON—deoxynivalenol; DHEA—dehydroepiandrosterone; cnt—control; *ANX5A*—annexin V.

**Table 4:** Primers used in RT-qPCR. This is a table. RPS17—ribosomal protein S17; RPLP0—ribosomal protein P0; H3F3A—histone H3.3A; CYP11A1—Cytochrome P450 Family 11 Subfamily A Member 1; CYP17A1—Cyto-chrome P450 Family 17 Subfamily A Member 1; CYP19A1—Cytochrome P450 Family 19 Subfamily A Member 1; HSD3B2—3 Beta- And Steroid Delta- Isomerase 2; HSD17B2—Hydroxysteroid 17-Beta Dehydrogenase 2; StAR—Steroidogenic acute regulatory protein; ANX5A—Annexin V, ESR1—estrogen receptor 1; ESR2—estrogen receptor 2; AR—androgen receptor.

Gene	Sequence	Product Size (bp)
*ANX5A*	For ACCCTCTCGGCTTTATGATGCT Rev TGGCTCTCAGTTCTTCAGGTGT	116
*AR*	For GGGAGGTTACACCAAAGGGC Rev AGAGACAGGGTAGACGGCAG	102
*CYP11A1*	For CCAGAACGATTCCTCATCC Rev CATCACCTCCTGGTTCAG	126
*CYP17A1*	For GAAGTTATCATCAATCTGTGGG Rev ACTGACGGTGAGATGAGC	119
*CYP19A1*	For CCTTCTGCGTCGTGTCATG Rev AAGATGTCTGGTTTGATGAGGAG	135
*ESR1*	For ATCTCGGTTCCGCATGATGAATCTGC Rev TGCTGGACAGAAATGTGTACACTCCAGA	98
*ESR2*	For ACACCTGGGCACCTTTCTCCTTTA Rev TCTTGCTTCACACCAGGGACTCTT	90
*HSD3B2*	For CTTGGTGTCACTCACAGAGAG Rev GTAGATGAAGACTGGCACACTG	128
*HSD17B2*	For TCTCTACTCCATGTACTCAG Rev CACCTCCAATTGTGACATAA	218
*H3F3A*	For AGGACTTTAAAAGATCTGCGCTTCCAGAG Rev ACCAGATAGGCCTCACTTGCCTCCTGC	74
*RPLP0*	For ACGGATTACACCTTCCCACTTGCTAAAAGGTC Rev AGCCACAAAGGCAGATGGATCAGCCAAG	69
*RPS17*	For AAGCGCGTGTGCGAGGAGATCG Rev TCGCTTCATCAGATGCGTGACATAACCTG	87
*STAR*	For CATGGAGAGGCTCTATGAAGA Rev CAGCCAGCTCGTGAGTAAT	128

## Data Availability

Most of the data are presented in the study. The data not presented in this study are available on request from the corresponding author.
